# A methodology for estimation of land use changes in an urban area with the emergence of a new impact factor

**DOI:** 10.1016/j.mex.2020.101013

**Published:** 2020-07-27

**Authors:** Torkan Borna Seifloo, Mehmet Ali Yuzer

**Affiliations:** Department of Urban and Regional Planning, Faculty of Architecture, Istanbul Technical University, Harbiye Mahallesi, Taskisla Cad., 34367 Sisli, Istanbul, Turkey

**Keywords:** Land use changes, Land-use assessment model, Sieve analysis, Natural environmental factors, Socio-economic factors, Quantitative evaluation model, Airport effect, Urban development

## Abstract

This article introduces a methodology that aims to develop a detailed framework for measuring land-use changes in an area affected by several impact factors, especially by a new project which is economically and spatially critical in an urban area. The basis of the methodology lays on the Sieve approach that analyzes changes by categorizing different factors in an urban settlement or even on vacant lands. It is a multi-factorial approach that evaluates and simulates land-use changes in an urban area with a newly-emerged impact factor (Airport in this study). Factors such as proximity to Transportation nodes, Commercial areas, Industrial areas, Health centers, Educational areas, and many others that define investors, users, and planners' tendencies are all critical parameters that can be used as inputs of this quantitative approach.

Function Impact Analysis Oriented Land-Use Assessment Model (APELUM), developed in this study, is a new method useful for measuring land cover changes in an urban area even before it takes place. It can be used as a method for analyzing different scenarios to determine the strengths and weaknesses of each model. Two different scenarios, supported by two different spatial models, are evaluated: "Demands and trends of the users and investors in the urban area" that simulates rapid and unplanned development and “The planning decisions and user trends" to simulate strategic development.

The methodology uses AutoCAD and ArcGIS tools for producing maps and making different measurements.

• This quantitative model and similar ones are useful tools for measuring the range of land-use changes after or before the implementation of a new project in an urban area.
• It generates quantitative estimations based on spatial data in the planning process of cities.
• It can be useful to develop simulation models that make correlations between different features of measured tendencies.

## Graphical abstract

**Figure:**
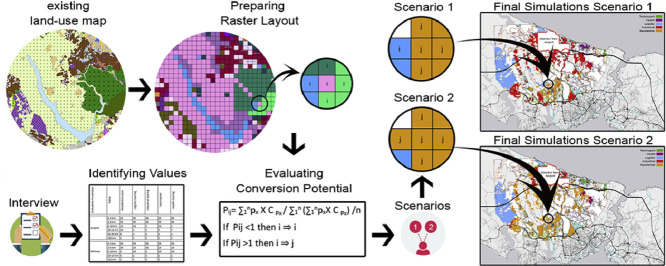

Specifications Table

**Table:** 

Subject Area:	Environmental Science
More specific subject area:	Airport development effects, land-use tendencies
Method name:	Airport effective land use estimation model (APELUM)
Name and reference of original method	
Resource availability	

## Method details

### Basis of the concept

Geographers have consistently focused on network studies as noteworthy variables of urban growth, while different analysts have mostly paid attention to airport-related factors. American urban specialist John Kasarda has conspicuously looked for Airport-Led Urban Development. He argues that airports are noteworthy components of forming urban space within the twenty-first century, much as highways did within the twentieth century, railroads within the nineteenth century, and seaports within the eighteenth century. He proposes that this innovative wave of transportation impacts fundamentally through commercial, logistics, and other financial activities. Kasarda has been noticeable in keeping up the noteworthiness of airport terminals as prominent factors for nearby and territorial economic improvements and land-use changes. The boundary between airports and their domain has continuously been covered with commercial, hotel, residential, industrial, and logistic complexes immediately after the opening. In most cases, airport-led improvements have been unconstrained and poorly-planned missing arranging vital advancements [3].

In the last two decades, numerous modern urban development models have been created that address distinctive urban issues. The most significant part of them are based on particular models, such as Environmental modeling, Land use &Transportation (LUT) models, Cellular Automata-based models (CA), Agent-based models (ABMs), Integrated models, and Urban innovative financial models. Although their connections are dismissed, the majority of existing surveys utilize LUCC modeling as their starting focuses [9].

The method described in this article is supported by comprehensive programs developed with the increase in computers' capacity. The methodology uses AutoCAD and GIS programs for producing maps and making different measurements. The model, which will be explained in detail in this article, is a Sieve analysis-based model, which is frequently used in planning. Sieve analysis mainly provides results that generate data at the synthesis stage during the planning process [1]. The method developed in this study (APELUM), is a new method useful for measuring land cover changes even before the changes take place. It can be used as a method for analyzing different scenarios to determine the strengths and weaknesses of each model. Various unlimited impact factors can be measured to evaluate their effects on the land cover. Different scenarios also can be considered to compare the positive and negative aspects of each analysis.

However, the model conveyed in this article can predict urban land-use changes based on various assumptions and perspectives. Therefore, before starting the analysis of the model based on impact factors, which constitutes the essence of this article, it is crucial to give the chronological development process of the studies with similar approaches. Two approaches have been adopted to the study in determining the changes in land-use planning. The development processes of these approaches and related models are as follows;

• Models that determine the range of change

The primary purpose of these models is to estimate the possible effects of the defined areas based on the interactions between transportation and land use. In this regard, after the ''Detroit Urban Simulation Model'' which was a metropolitan Transportation Model developed in 1955–1956, the ''Chicago Area Transportation Study'' started to grow in the 1960s. These computer-based models were intended to increase transportation speed in the metropolitan area using active transportation layouts. In the 1960s, a prototype model based on linear programming was developed by Herbert-Stevens in the development estimation of residential regions. The beginning of the "Push type" model designed for land use predictions based on the work published in 1961 by the “Lowry Model” [1].

• Land-use forecasting models

The "Land-Use Estimation Models" have evolved into visualization with the development of computers and programming models. In this context, Portugali and Benenson generated the global organization regulations with the help of CA models by applying the general definition to the multidimensional presentation of cultural identity we define local spatial cognitive dissonance in 1997 [10]. City simulation models that estimate CA-based urban development and transformation using the combination of theoretical concepts and experimental facts were developed by White and Engelen at the same year [1]. "Reading Model" developed by Batty in 1973 and "Multipurpose Land-Use Model" was developed by Cagdas, Dokmeci, and Tokcan in 1988 [8]. The CA-based LUCAM model, which allows simulation of urban transformation and development was introduced in 2000 [4]. Rapidly developing high-capacity computer technology and programs have allowed the development of new models similar to those given above.

### Istanbul airport

#### Existing land use of the airport neighborhood

The western part of Istanbul, which covers 20 km from the airport, includes land uses such as Vacant land, Forest areas, Passive green areas (green areas that are abandoned from construction and physical activity), Water Basins, Agricultural areas, and partially Rural residential areas. Istanbul Airport has been planned and designed not only as a passenger terminal but also as one of the essential business centers to serve in Istanbul. With the planning of the airport, the construction of the 3rd Bosphorus Bridge, and the 3rd Ring Road Project and with the development of the Istanbul transportation system, the attraction and impact of the airport will increase rapidly. It will lead to the rise in the neighborhood land values, the development trends of the industrial sectors trapped in the city center, which have no enough space for growth, and the need for the rapidly growing industries such as logistics and technology centers. Furthermore, the possible workforce which will work in these sectors is a significant factor that brings the necessity of other land uses such as residential, health centers, educational areas, and commercial areas to serve the population.

The interaction of Istanbul airport with the Ring Road project, the third Boğaz bridge, and other developing projects and its proximity to the water basins, natural areas (forests, agricultural fields, etc.) and passive green areas has been a controversial issue among the central government, the local government, the academic circles, NGOs, investors, and Istanbul residents for a long time [4]. In this study, with the construction of Istanbul Airport, potential land-use changes in the region will be evaluated and simulated.

### The procedure

The study uses APELUM base on Sieve Analysis methodology which is implemented in the analyses of Istanbul Airport neighborhood, as the case study.

The methodology consists of several steps (Table 1) that are organized in an incremental process. In the first step fundamentals of the simulation model will be defined. In this study, two scenarios are defined as the first step of the study in order to compare airport effective urban development directed by the demands and trends of the users and investors in the urban area as a schema of rapid and unplanned development and simulation does urban development, where planning decisions and user trends are effective.

**Table 1:** Summary of all stages processed throughout the simulation.

**Fig. 1:**
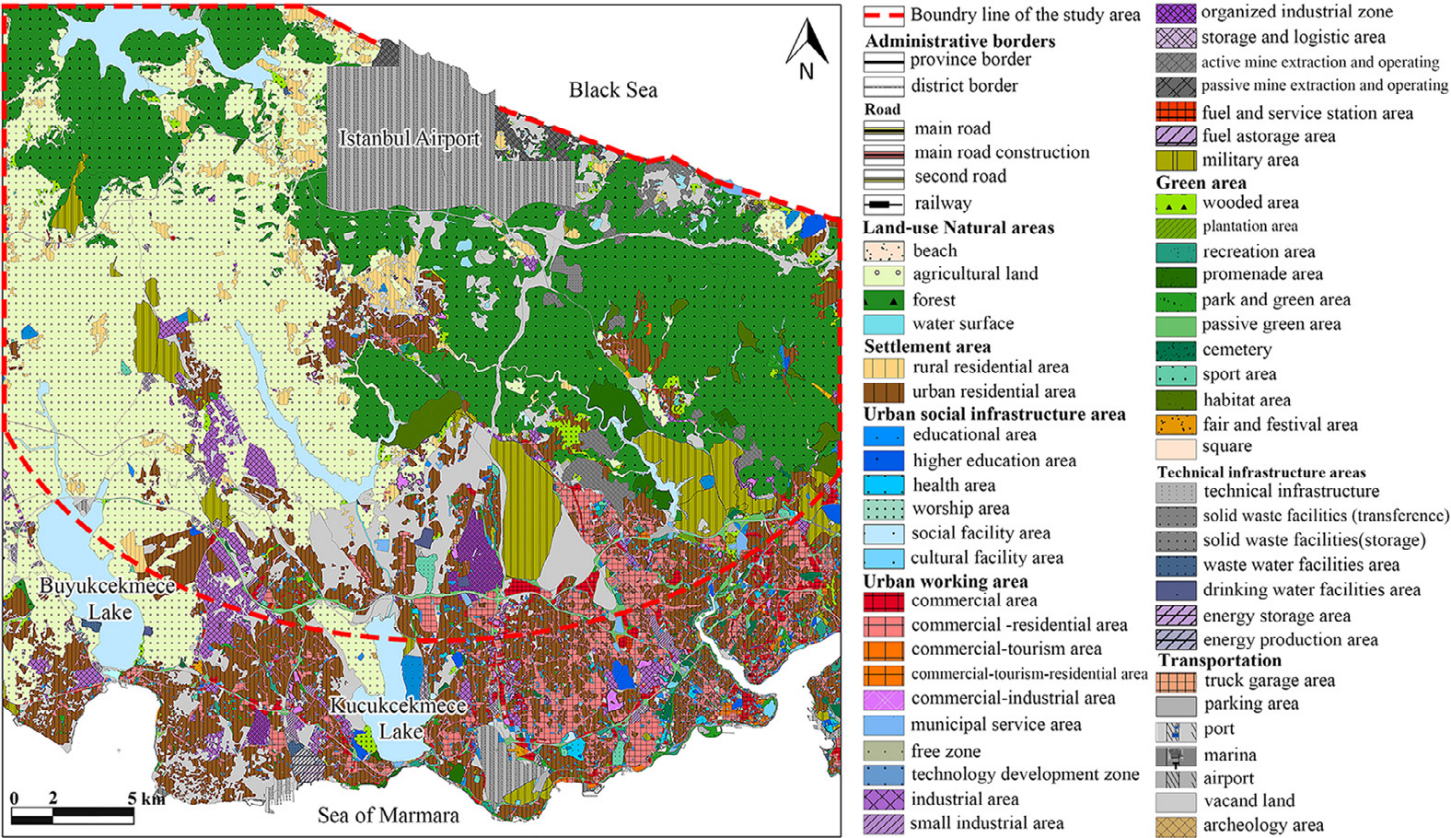
Land use planning map of the study area [7].

**Fig. 2:**
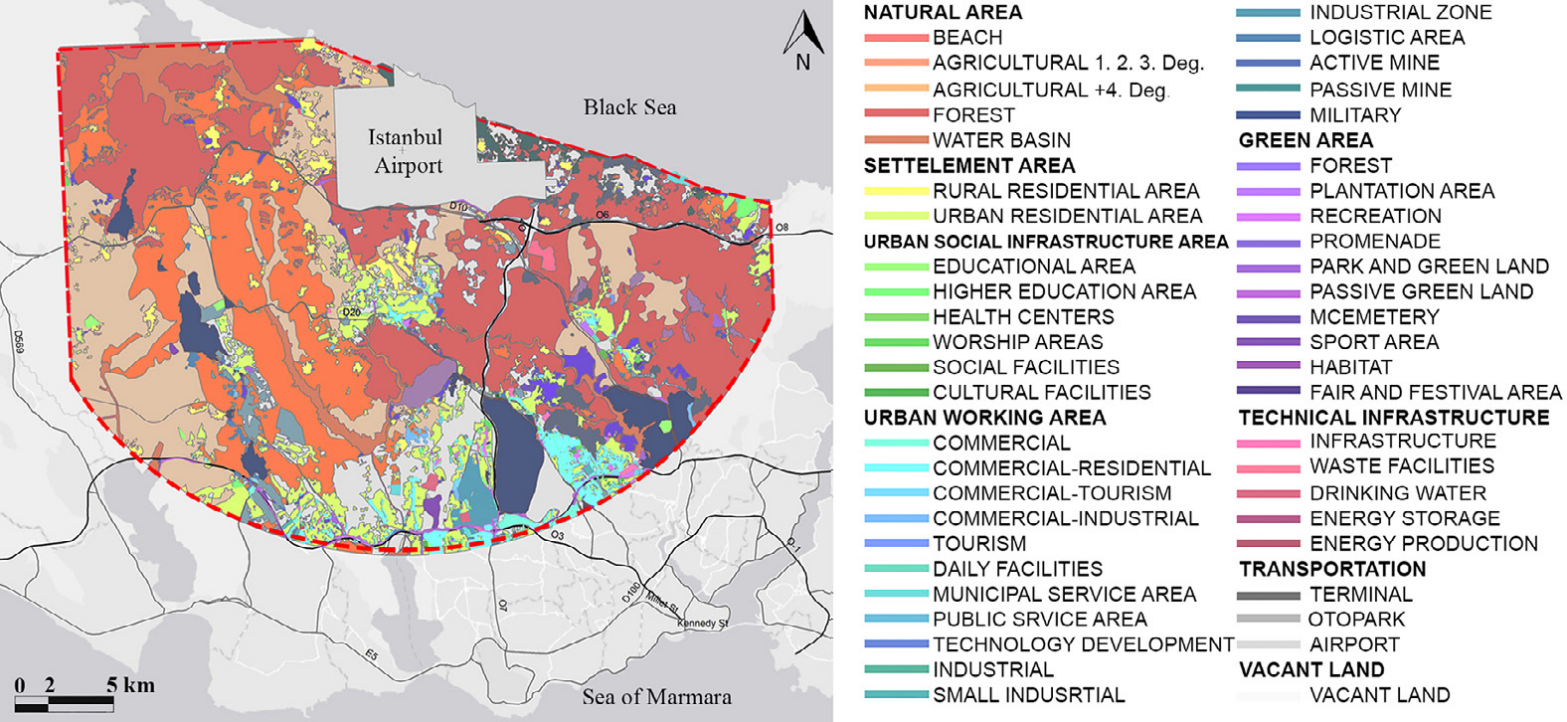
Classified land uses of the case study area in ArcGIS program.

AutoCAD and ArcGIS programs are used for making different data measurements. Firstly, the 1/50.0000 scale map showing Istanbul airport and its 20 km surrounding which was released by Istanbul Municipality (Fig. 1) was used as a layout to categorize different land uses in different layers in AutoCAD: Natural area, settlement area, Infrastructure area, Working area, Green Area, Transportation, and Vacand land. After that, the layers are exported to ArcGIS separately. Each of the layers is converted in shape files in ArcGIS (Fig. 2).

**Fig. 3:**
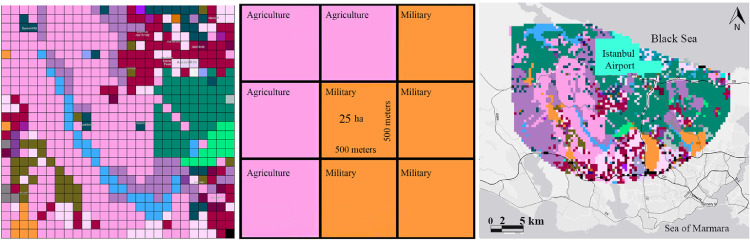
Identification of ID object to the classified land uses for the cells of 25 hectares.

Secondely, the detailed map (in the previous stage) of Istanbul is divided into grids of 500 m x 500 m in the ArcGIS environment. An "Object ID" is defined for each cell (Fig. 3). After these arrangements, the evaluations begin to process which is explained in detail in 7 steps.

Assessing a model based on impact factors which aim to evaluate all data in the planning process and to estimate the future land use is structured according to five objectives and tendencies. In determining spatial development tendencies around the airport following factors are considered as general parameters in creating future-oriented scenarios following guidelines;

• Sustainability of that natural resources
• Social trends and expectations
• The effects of the transportation system on land use
• Different sector's expectations and tendencies
• Spatial impacts of demographic and spatial projections

Questionnaires were conducted to reveal these differences and determine the preferences of the user's and invester's trends. In estimating the development processes of the cities, making a scenario based on the sustainability of the natural structure without considering the trends, needs, and expectations of the investors or users makes the policy and plan decisions to be developed incomplete for the future of the city. Likewise, an assessment that defines urban development only as a response to the user and investor trends, and does not care about the sustainability of the natural structure, will negatively affect urban development.

In this article, the basic setup of assessing the model based on impact factors, simulates how the significant functions in the city may affect land-use decisions in the neighborhood context (20 km radius of the airport). The model consists of 7 stages (Table 1).

100 interviews conducted with the residential users which are evaluated based on the responses. Each criterion is valued over 100 according to the results. The coefficient values of the parameters are determined according to the answers.

All answers are valued over 100 (Table 6) based on the evaluation parameter values (P_x_) which are proposed for rating to the interviewees. While scoring, users’ tendency are compared with experts’ opinion (City and Regional _P_lanners, Architects, Engineers, and other relevant technicians) to determine the impact value for each parameter (Table 7).

**Table:** 

Fundamentals of the simulation model will be defined in this stage. In this study, two scenarios were structured. The First one is structured based on users, sector representatives' preferences, and expert opinions evaluation. The second one is based on users, sector representatives' preferences, the assessment of experts’ views, and the projections of the 2009 Istanbul Environmental master Plan.

The general principle of Scenario 1 and Scenario 2 approaches: Planning strategies should be used to determine the development of settlements' development. Especially in developed countries and/or in countries where planning studies give direction to urban development. While making future decisions, all stages of the city planning discipline (goals, objectives, analysis, synthesis, strategy development, making a selection among alternative developments, etc.) are carried out. In some developing countries, "urban development" completes the process before "planning activities." Improvements can take place in a region without planning and according to user trends and expectations. Since the 1950s, due to rapid urbanization, urban development has always been irregularly ahead of the planning actions in Istanbul. The effectiveness of planning decisions has increased in the determination of urban development areas since the 1990s.

In this study, In Scenario 1, it is aimed to determine the possible airport effective urban development scheme, which is effectively directed by the demands and trends of the users and investors in the urban area. The goal in this scenario is to simulate rapid and unplanned development. In this study, the second scenario aims to determine the possible airport effective urban development scheme in the cities, guided by both the user and investor requests and the decisions taken after the planning activities. In this scenario, the aim is to simulate urban development, where both planning decisions and user trends are effective.

**Table:** 

Land use and transportation data of the natural and the built-up environment are classified and transferred to a digital environment using the ArcGIS program. At this stage of the model, the 1/50.0000 scale map showing Istanbul airport and its 20 km surrounding were prepared based on the data released by the Municipality.

Land use and transportation data of the natural and the built-up environment are classified and transferred to a digital environment using the ArcGIS program. At this stage of the model, the 1/50.0000 scale map showing Istanbul airport and its 20 km surrounding were prepared based on the data released by the Municipality. In this stage, data related to various functions, such as natural land uses, built-up areas, vacant land, and transportation data (roads, railways, intersections) [5] are classified.

The detailed map (in the previous stage) of Istanbul is divided into grids of 500 m x 500 m in the ArcGIS environment. An "Object ID" is defined for each cell.

**Table:** 

The effect that each function will have on the model is defined numerically. User tendencies and expectations and expert opinions are evaluated in determining the impact values of the features. A survey is conducted in residential areas with those who financially support their family. Neighborhoods are randomly selected in the urban area inside the case study boundary, to identify 100 users' tendencies and expectations.

In addition, in-depth interviews are held to determine the trends and expectations of sector representatives. Related representatives are selected from the top-ranked private organizations and governmental institutions in Istanbul.

In the simulation process, to assess the impact assessment of the effect that each function will have on the model is defined numerically (Table 6). User tendencies, expectations, and expert opinions are evaluated in determining the impact values of the functions (Table 7). It is assumed that the number of surveys for Residential assesssment will be sufficient to determine user trends as;

• The population of Istanbul in 2019 is: 15,519,267
• Istanbul European Continent Population: 9,726,373
• The average family population: 3.4 people
• estimated number of families in the European Continent of Istanbul: 2,800,000
• The number of survey samples is 100/2.800.000 = 1 in 28.000 [2].

For the simulation of the study area in addition to the residents, five In-depth interviews and surveys (Totally 20) were conducted with leaders of each sector (Logistics, Industry, Techno park, and Health). It was assumed that in-depth interviews and surveys with sector representatives would be more effective in determining trends rather than performing a large number of random surveys.

**Table:** 

All interviews conducted with the residential users are evaluated based on the responses. Each criterion is valued over 100 according to the results. The coefficient values of the parameters are determined according to the answers. For the simulation of the study area in addition to the residents, five in-depth interviews and questionnaires were held with the Logistic, Industrial, Technoparks, and Health sectors’ representatives. All answers are valued over 100. While scoring, users’ tendency and expectations are compared with experts’ opinions (City and Regional Planners, Architects, Engineers, and other relevant technicians). Experts always consider criteria such as sustainability of the natural structure, Socio-Economic balance, and development, urban development and transportation connections, inter-functional interaction, planning principles, public interest, the relationship and effects of airports with the nearby environment [11].

**Table:**

**Table:**

**Table:**

**Table:**

**Table:**

**Table:** 

The final stage of the model is to determine the possible future land use of the settlements after the simulation, to draw the first and second schemes.

The following table represents a sample evaluation of a cell that calculates conversion value from its current land use to the logistic use. For the considered function evaluation (logistic), the values that each cell takes from the specified parameters are determined (p_x_) and multiplied by the Influence values (Coefficient value) of the parameters (*C_px_*). These values are summed to obtain the overall transformation value of the cell (*C _tmv_*). All these processes are applied to other cells, respectively, to achieve the total transformation value of all cells (∑^1^*_n_ C _tmv_*). Intermediate transformation value is calculated by dividing this value by the total number of cells. In the final step, the transformation value of each cell is divided by the cells’ Intermediate transformation Value (*C _tmv_*/*C_tv_*). If the value (*P_ij_*) is greater than 1, it turns into the considered function; otherwise, it remains in its old function (Table 2).

**Table 2:** The calculation process of a cell's Conversion possibility to logistic land use.

Object ID	Distance from the Airport	Railway	Intersection	Industrial	Land value	Educated worker	Water basin
Values (*p_x_*)	39	19	19	1	95	38	20
Influence value (*C _px_*)	15	12	10	20	12	12	12
*p_x_*x *C_px_*	741	228	190	20	1140	456	240
transformation value (*C _tmv_*)	3015						
Total transformation Value of all cells (∑_1_*^n^ C _tmv_*)	12,796,134						
The Total number of evaluated cells (*n*)	4258						
Intermediate transformation Value *C _tv_*	12,796,134/4258 = 3005						
Conversion Value from one function (*i*) to another Function (*j*) *P_ij_*	3015/3005= 1.003 So *P_ij_*>1 then *j*= Logistik						

### Simulation model of the study area

**Fig. 4:**
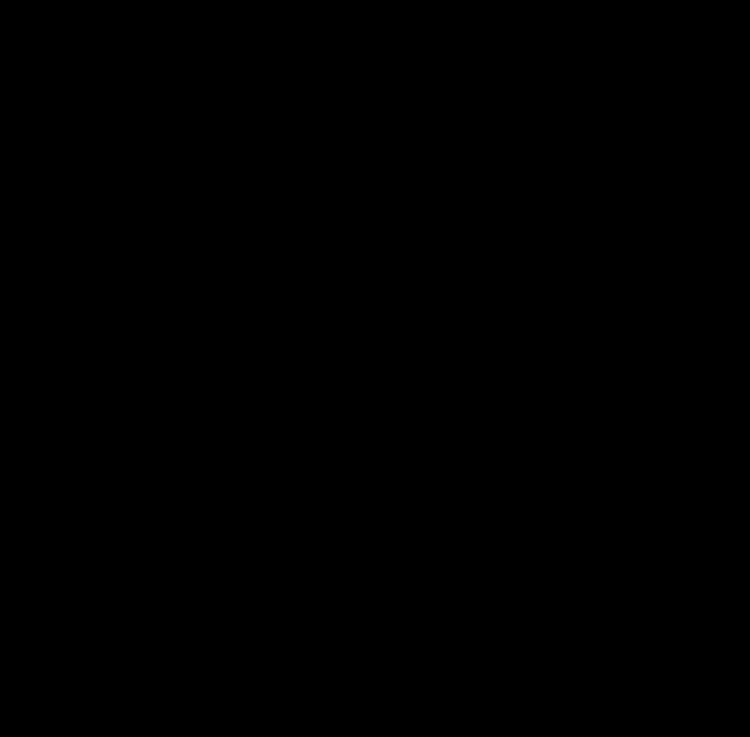
Conversion simulation model of each function. (For interpretation of the references to color in this figure, the reader is referred to the web version of this article.)

The model is simulated on an area of 110,500 hectares on Istanbul airport neighborhood in the west part of the city. According to user tendencies and expectations of the sector representatives, all calculations are made (as described in the previous section). The transformation value of the cells with vacant and 4th grade and higher agricultural land use (4th grade and higher agricultural areas are areas construction is allowed based on legislation) into the residential, Logistic, Health, Industrial, and Technopark are evaluated and simulated, respectively. As a result of the evaluation, cells that were simulated in yellow, blue, purple, red, and green appear as cells prone to transformation (Fig. 4).

**Fig. 5:**
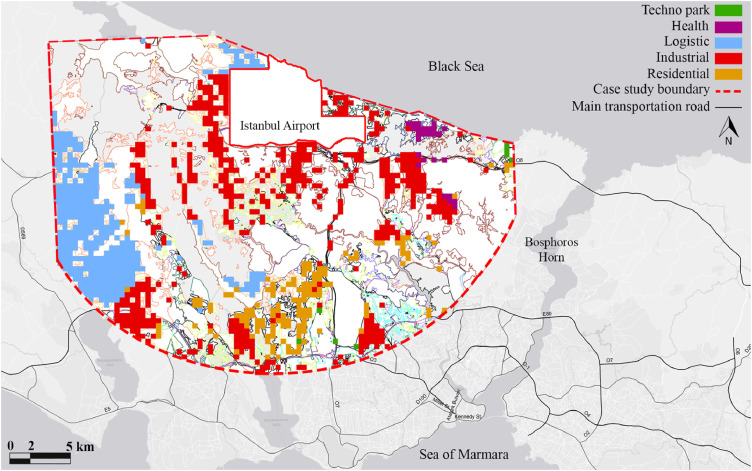
Residential, logistics, industry, health cell simulation model according to the users' preferences, the first scenario scheme.

As the final evaluation of the first scenario, the values of each cell prone to transformation are compared to find out the function from which the cell has achieved a maximum amount. The cells that reach the highest values are transformed to the related function and simulated (Fig. 5).

The second scenario is based on planning strategies and tendencies of the Istanbul Metropolitan Municipality. The coefficient values in this scenario are determined according to the land use ratio presented in the Istanbul master plan report in 2009 (Table 3). Existing land use distributions are presented in percentage in the “characteristic table”. Istanbul Population is accepted as 16 million people with an area of 540,000 hectares.

**Table 3:** Urban character table, environmental planning report, Istanbul Municipality, 2009[6].

Existing Land use	%
logistic	0,3
Industrial	0,9
Techno parks	0,5
Residential	12,1
Health	0,2

New coefficients for the second scenario are calculated based on data provided by the municipality (Table 4).

**Table 4:** Coefficient values of the second scenario.

Evaluated Land uses	%
logistic	0,4
Industrial	0,9
Techno parks	0,5
Residential	8
Health	0,2

The following table summarizes area variations of the existing land uses and the land use areas of the first and the second scenarios.

Considering the land-use distribution based on land area, the current land use that covers the maximum area and tends to develop in the airport neighborhood is Residential with 10,023 ha. The maximum land cover is Industrial with 12,125 ha in the first scenario analysis and residential with 14,500 ha in the second scenario analysis (Table 5).

**Table 5:** Summary table of the evaluated land uses.

Land Use	Current area (ha)	Simulation 1 area (ha)	Simulation 2 area (ha)
Residential	10,023	13,673	24,523
Logistic	287	8987	7962
Industrial	2272	14,397	3797
Health	5,7	505,7	555,7
Techno parks	11,74	211,74	936,74
4th grade and higher agricultural area	27,070	1895	1895
Vacant Land			
Others	72,013	72,013	72,013
Total	111,683	111,683	111,683

**Table 6:** Scoring system based on users’ tendencies.

**Table 7:** Parameters’ coefficient values calculation table.

Evaluation criteria	Land use	Total of the highest values *C_pQ_*	User preferences *C_pµ_*	Expert view *C_pe_*	Influence value *C _px_*
Airport	Residential	60	8	7	8
	Logistic	79	13	15	15
	Industrial	96	16	15	15
	Health	96	16	13	15
	Techno parks	78	12	14	16
					
Workplaces	Residential	60	8	10	9
					
Railway	Residential	88	12	9	10
	Logistic	78	13	12	12
	Industrial	96	16	14	13
	Health	78	13	12	12
	Techno parks	78	13	13	14
					
Active green land	Residential	82	11	8	10
					
Intersections	Residential	62	9	7	8
	Logistic	96	16	10	13
	Industrial	96	16	15	15
	Health	96	16	13	14
	Techno parks	78	13	14	15
					
Educational	Residential	30	5	9	7
					
Health	Residential	65	9	8	9
					
Socio-Cultural	Residential	50	7	7	7
					
Commercial	Residential	70	9	11	10
	Health	96	16	15	15
					
Land value	Residential	70	9	12	10
	Logistic	96	16	12	14
	Industrial	96	13	14	15
	Health	96	16	12	14
					
Rent Prices	Residential	60	8	11	10
					
Water basin	Logistic	64	11	11	11
	Industrial	64	10	10	10
	Health	64	10	10	10
	Techno parks	64	11	12	11
					
Educated worker	Logistic	96	16	12	14
	Industrial	77	13	13	13
	Health	96	16	12	14
					
Other Industrial	Logistic	78	13	20	16
	Industrial	96	16	16	16
	Techno parks	58	12	14	13
					
Hotel	Health	78	13	13	13
					
Techno parks	Techno parks	58	10	16	13
					
Universities	Techno parks	96	16	17	17

**Fig. 6:**
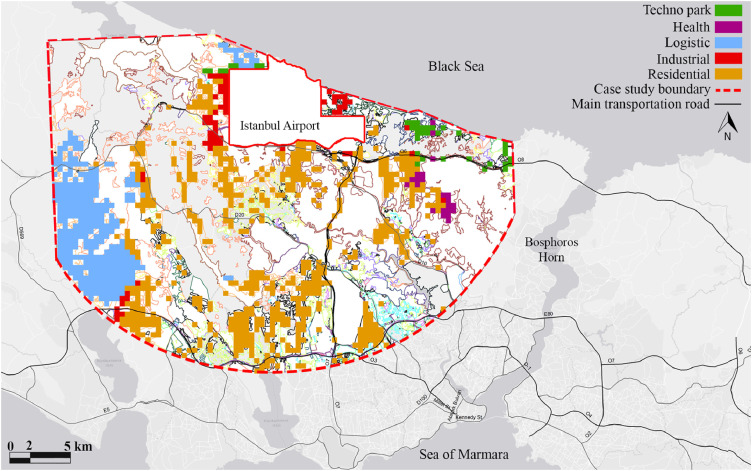
Residential, logistic, industry, and health cell simulation model according to the land-use planning strategies projected by Istanbul Metropolitan Municipality, the second scenario scheme.

The first scenario developed based on user tendencies demonstrates that the Industrial sector has the highest propensity to develop in the airport neighborhood which will lead to a rapid and an unplanned development. The second scenario shows future strategic projections and expectations of the municipality that foreseen residential developments more than other land uses (Fig. 6).

Different Scenarios can be measured with this method by evaluating various indicators to measure users, investors, urban planners, and authorities' expectations and tendencies. In this study, the impact factors are measured by in-depth interviews with sector leaders rather than performing a large number of random surveys as it provides more unforeseen problems. Although It may be complicated, it gives an accurate database to determine sector representatives' trends. A large amount of quantitative data is analyzed in ArcGIS to simulate different scenarios by importing various and interconnected inputs. Moreover, it is mandatory to access the latest land-use data, issued by authorities, to make a comparative evaluation of the land-use changes in an area

## Supplementary material

### Survey questions

- The effect of being close to the airport, workplaces, metro stations, active green areas, transportation intersections, educational areas, health facilities, socio-cultural facilities, shopping centers-commercial facilities has been questioned according to the distance.
- The impact of land costs and/or housing prices were questioned in monetary value ranges.
- The effects of rental fees were questioned in monetary value ranges.

Surveys and interviews also conducted for other functions;

Questions were asked in the primary headings. The issues of the residential survey were also asked to the other sectors. Besides, some follow up questions were also added:

- Name surname/Duty/Core activity/Workplace area (m2)
- Property Status: Owner/Tenant
- The number of employees:
- Workplace Function: Central branch/storage/showroom/factory

Please list the following factors in order of importance;

**Table:** 

Parameters	Order of importance	Specify Value (km/number/price)
Number of workers (The number is presented to the sector representatives during the survey based on the data received from the Ministry of Population and Citizenship Affairs)		
Proximity to other suppliers		
Land value (Presented to the investor during the survey according to the data from the Price Index)		
The population of educated workers		
Proximity to hotels		
Proximity to Universities		
Proximity to other similar sectors

- Are you considering moving your workplace or opening other branches?
- If yes, Where do you plan to move?
- What kind of benefits does proximity to the airports provide for your company?
- What kind of benefits does the proximity to the Istanbul New airport offers for your company?
- How far do you prefer to be from the airport in your future location selections?
- Which region is your most profitable investment?
- In which areas your company have invested until now?

## Declaration of Competing Interest

None.

## Appendix Supplementary materials

Image, application 1

## References

[1] Yuzer M.A. (2011). Quantitative data evaluation model in the process of planning: case of Istanbul Metropolitan Area. AZ ITU J. Fac. Archit..

[2] Governorship of Istanbul Republic of Turkey, 2019 Report.

[3] Freestone R., D Baker (2011). Spatial planning models of airport-driven urban development. Sage J..

[4] Yuzer M.A. (2000). Determination of Urban Development Areas By Fractal And Cellular Automata Method.

[5] Istanbul Metropolitan Municipality, Metro Line Development Plan, 2020

[6] Istanbul Metropolitan Municipality, 100/000 Environmental Plan Report, 2009

[7] BIMTAS Bogazici Construction Consultancy Inc., Land Use Development Master Plan, 2010

[8] Dokmeci V. (2015). Numerical Methods in Planning.

[9] L Xuecao, Gong P. (2016). Urban growth models: progress and perspective. Sci. Bull. J..

[10] Portugali J., Benenson I., Omer I. (1997). Spatial cognitive dissonance and socio-spatial emergence in a self-organizing city environment and planning B. v24.

[11] Dede O. (2016). The Analysis of Turkish Urban Planning Process Regarding Sustainable Urban Development.

