# Supplementary material for: A methodology for estimation of land use changes in an urban area with the emergence of a new impact factor
Source: MethodsX. 2020 Jul 27;7:101013. doi: 10.1016/j.mex.2020.101013 (PMC7415929; doi:10.1016/j.mex.2020.101013)
Supplement: Supplementary file 1 [file mmc1.docx]

**Supplementary material**

*Survey Questions*

- The effect of being close to the airport, workplaces, metro stations, active green areas, transportation intersections, educational areas, health facilities, socio-cultural facilities, shopping centers-commercial facilities has been questioned according to the distance.
- The impact of land costs and/or housing prices were questioned in monetary value ranges.
- The effects of rental fees were questioned in monetary value ranges.

Surveys and interviews also conducted for other functions;

Questions were asked in the primary headings. The issues of the residential survey were also asked to the other sectors. Besides, some follow up questions were also added:

- Name surname/Duty/Core activity/Workplace area (m^2^)
- Property Status: Owner/Tenant
- The number of employees:
- Workplace Function: Central branch/storage/showroom/factory

Please list the following factors in order of importance;

| Parameters | Order of importance | Specify Value (km/number/price) |
| --- | --- | --- |
| Number of workers (The number is presented to the sector representatives during the survey based on the data received from the Ministry of Population and Citizenship Affairs) |  |  |
| Proximity to other suppliers |  |  |
| Land value (Presented to the investor during the survey according to the data from the Price Index ) |  |  |
| The population of educated workers |  |  |
| Proximity to hotels |  |  |
| Proximity to Universities |  |  |
| Proximity to other similar sectors |  |  |

- Are you considering moving your workplace or opening other branches?
- If yes, Where do you plan to move?
- What kind of benefits does proximity to the airports provide for your company?
- What kind of benefits does the proximity to the Istanbul New airport offers for your company?
- How far do you prefer to be from the airport in your future location selections?
- Which region is your most profitable investment?
- In which areas your company have invested until now?

**Table 6**

Scoring system based on users’ tendencies

| Evaluation Parameter | Value (p_x_) | Residential value (p_x_) | Logistic Scoring (p_x_) | Industrial Scoring (p_x_) | Technopark (p_x_) | Health Scoring(p_x_) |  | Evaluation Parameter | Value | Residential Scoring (p_x_) | Logistic Scoring (p_x_) | Industrial Scoring (p_x_) | Technopark Scoring(p_x_) | Health Scoring (p_x_) |
| --- | --- | --- | --- | --- | --- | --- | --- | --- | --- | --- | --- | --- | --- | --- |
|  |  |  |  |  |  |  |  |  |  |  |  |  |  |  |
| Airport | 0-3 km | 15 | 39 | 38 | 19 | 38 |  | Socio-Cultural | 0-5 km | 10 | - | - | - | - |
|  | 3-6 km | 55 | 39 | 58 | 59 | 58 |  |  | 5-10 km | 40 | - | - | - | - |
|  | 6-10 km | 15 | 19 | 1 | 19 | 1 |  |  | 10-15 km | 24 | - | - | - | - |
|  | 10-15 km | 10 | 1 | 1 | 1 | 1 |  |  | 15-20 km | 20 | - | - | - | - |
|  | 15-20 km | 4 | 1 | 1 | 1 | 1 |  |  | 20-25 km | 5 |  |  |  |  |
|  | >20 km | 1 | 1 | 1 | 1 | 1 |  |  | >25 km | 1 |  |  |  |  |
|  |  |  |  |  |  |  |  |  |  |  |  |  |  |  |
| Railway | 0-3 km | 50 | 59 | 58 | 59 | 59 |  | Commercial | 0-5 km | 40 | - | - | - | 58 |
|  | 3-6 km | 39 | 19 | 38 | 19 | 19 |  |  | 5-10 km | 30 | - | - | - | 38 |
|  | 6-10 km | 5 | 19 | 1 | 19 | 19 |  |  | 10-15 km | 27 | - | - | - | 1 |
|  | 10-15 km | 5 | 1 | 1 | 1 | 1 |  |  | 15-20 km | 2 | - | - | - | 1 |
|  | >15 km | 1 | 1 | 1 | 1 | 1 |  |  | >20 km | 1 | - | - | - | 1 |
|  |  |  |  |  |  |  |  |  |  |  |  |  |  |  |
| Intersections | 0-3 km | 35 | 39 | 78 | 59 | 38 |  | Housing land value | <250.000 | 40 | - | - | - | - |
|  | 3-6 km | 35 | 39 | 18 | 19 | 58 |  |  | 250.001-450.000 TL | 30 | - | - | - | - |
|  | 6-10 km | 20 | 19 | 1 | 19 | 1 |  |  | 450.001-600.000 TL | 18 | - | - | - | - |
|  | 10-15km | 9 | 1 | 1 | 1 | 1 |  |  | 600.001-750.000 TL | 7 | - | - | - | - |
|  | >15 km | 1 | 1 | 1 | 1 | 1 |  |  | >750.001 TL | 5 | - | - | - | - |
|  |  |  |  |  |  |  |  |  |  |  |  |  |  |  |
| Educational | 0-5 km | 5 | - | - | - | - |  | Health | 0-3 km | 10 | - | - | - | - |
|  | 5-10 km | 25 | - | - | - | - |  |  | 3-6 km | 55 | - | - | - | - |
|  | 10-15 km | 30 | - | - | - | - |  |  | 6-10 km | 10 | - | - | - | - |
|  | 15-20 km | 20 | - | - | - | - |  |  | 10-15 km | 10 | - | - | - | - |
|  | 20-25 km | 19 | - | - | - | - |  |  | 15-20 km | 14 | - | - | - | - |
|  | >25 km | 1 | - | - | - | - |  |  | >20 km | 1 | - | - | - | - |
|  |  |  |  |  |  |  |  |  |  |  |  |  |  |  |
| Water basin | 0-5 km | 1 | 1 | 1 | 1 | 1 |  | Rent prices | <1000 TL | 20 | - | - | - | - |
|  | 5-7 km | 15 | 15 | 15 | 15 | 15 |  |  | 1001-1500 TL | 40 | - | - | - | - |
|  | 7-10 km | 20 | 20 | 20 | 20 | 20 |  |  | 1501-2000 TL | 25 | - | - | - | - |
|  | 10-15 km | 29 | 29 | 29 | 29 | 29 |  |  | 2001-3000 TL | 10 | - | - | - | - |
|  | >15 | 35 | 35 | 35 | 35 | 35 |  |  | >3001 | 5 | - | - | - | - |
|  |  |  |  |  |  |  |  |  |  |  |  |  |  |  |
| Other Industrial | 0-5 km | - | 39 | 58 | 59 | - |  | Educated workers population | <50 P | - | 1 | 1 | - | 1 |
|  | 5-10 km | - | 39 | 38 | 19 | - |  |  | 70 p-90 p | - | 1 | 19 | - | 1 |
|  | 10-15 km | - | 19 | 1 | 19 | - |  |  | 90 p-110 p | - | 38 | 19 | - | 38 |
|  | >15 km | - | 1 | 1 | 1 | - |  |  | >110 p | - | 58 | 58 | - | 58 |
|  |  |  |  |  |  |  |  |  |  |  |  |  |  |  |
| Hotel | 0-3 km | - | - | - | - | 39 |  | Technopark | 0-3 km | - | - | - | 59 | - |
|  | 3-6 km | - | - | - | - | 39 |  |  | 3-6 km | - | - | - | 19 | - |
|  | 6-10 km | - | - | - | - | 19 |  |  | 6-10 km | - | - | - | 19 | - |
|  | >10 km | - | - | - | - | 1 |  |  | >10 km | - | - | - | 1 | - |
|  |  |  |  |  |  |  |  |  |  |  |  |  |  |  |
| Universities | 0-3 km | - | - | - | 58 | - |  | Commercial land value | <3800 TL | - | 95 | 58 | - | 58 |
|  | 3-6 km | - | - | - | 38 | - |  |  | 3800-4500 TL | - | 1 | 18 | - | 38 |
|  | >6 km | - | - | - | 1 | - |  |  | >4500 TL | - | 1 | 1 | - | 1 |
|  |  |  |  |  |  |  |  |  |  |  |  |  |  |  |
| Workplaces | 0-3 km | 55 | - | - | - | - |  | Active green land | 0-3 km | 52 | - | - | - | - |
|  | 3-6 km | 15 | - | - | - | - |  |  | 3-6 km | 30 | - | - | - | - |
|  | 6-10 km | 15 | - | - | - | - |  |  | 6-10 km | 17 | - | - | - | - |
|  | 10-15 km | 10 | - | - | - | - |  |  | >10 km | 1 | - | - | - | - |
|  | 15-20 km | 4 | - | - | - | - |  |  |  |  |  |  |  |  |
|  | >20 km | 1 | - | - | - | - |  |  |  |  |  |  |  |  |
|  |  |  |  |  |  |  |  |  |  |  |  |  |  |  |

**Table 7**

Parameters’ coefficient values calculation table

| Evaluation criteria | Land use | Total of the highest values  C_pQ_ | User preferences  C_pµ_ | Expert view  C_pe_ | Influence value  C _px_ |
| --- | --- | --- | --- | --- | --- |
| Airport | Residential | 60 | 8 | 7 | 8 |
|  | Logistic | 79 | 13 | 15 | 15 |
|  | Industrial | 96 | 16 | 15 | 15 |
|  | Health | 96 | 16 | 13 | 15 |
|  | Techno parks | 78 | 12 | 14 | 16 |
|  |  |  |  |  |  |
| Workplaces | Residential | 60 | 8 | 10 | 9 |
|  |  |  |  |  |  |
| Railway | Residential | 88 | 12 | 9 | 10 |
|  | Logistic | 78 | 13 | 12 | 12 |
|  | Industrial | 96 | 16 | 14 | 13 |
|  | Health | 78 | 13 | 12 | 12 |
|  | Techno parks | 78 | 13 | 13 | 14 |
|  |  |  |  |  |  |
| Active green land | Residential | 82 | 11 | 8 | 10 |
|  |  |  |  |  |  |
| Intersections | Residential | 62 | 9 | 7 | 8 |
|  | Logistic | 96 | 16 | 10 | 13 |
|  | Industrial | 96 | 16 | 15 | 15 |
|  | Health | 96 | 16 | 13 | 14 |
|  | Techno parks | 78 | 13 | 14 | 15 |
|  |  |  |  |  |  |
| Educational | Residential | 30 | 5 | 9 | 7 |
|  |  |  |  |  |  |
| Health | Residential | 65 | 9 | 8 | 9 |
|  |  |  |  |  |  |
| Socio-Cultural | Residential | 50 | 7 | 7 | 7 |
|  |  |  |  |  |  |
| Commercial | Residential | 70 | 9 | 11 | 10 |
|  | Health | 96 | 16 | 15 | 15 |
|  |  |  |  |  |  |
| Land value | Residential | 70 | 9 | 12 | 10 |
|  | Logistic | 96 | 16 | 12 | 14 |
|  | Industrial | 96 | 13 | 14 | 15 |
|  | Health | 96 | 16 | 12 | 14 |
|  |  |  |  |  |  |
| Rent Prices | Residential | 60 | 8 | 11 | 10 |
|  |  |  |  |  |  |
| Water basin | Logistic | 64 | 11 | 11 | 11 |
|  | Industrial | 64 | 10 | 10 | 10 |
|  | Health | 64 | 10 | 10 | 10 |
|  | Techno parks | 64 | 11 | 12 | 11 |
|  |  |  |  |  |  |
| Educated worker | Logistic | 96 | 16 | 12 | 14 |
|  | Industrial | 77 | 13 | 13 | 13 |
|  | Health | 96 | 16 | 12 | 14 |
|  |  |  |  |  |  |
| Other Industrial | Logistic | 78 | 13 | 20 | 16 |
|  | Industrial | 96 | 16 | 16 | 16 |
|  | Techno parks | 58 | 12 | 14 | 13 |
|  |  |  |  |  |  |
| Hotel | Health | 78 | 13 | 13 | 13 |
|  |  |  |  |  |  |
| Techno parks | Techno parks | 58 | 10 | 16 | 13 |
|  |  |  |  |  |  |
| Universities | Techno parks | 96 | 16 | 17 | 17 |
